# Magnetic structure in the two-dimensional van der Waals ferromagnet Fe_3_GaTe_2_

**DOI:** 10.1107/S160057672600230X

**Published:** 2026-05-08

**Authors:** Po-Chun Chang, Sabreen Hammouda, Yung-Hsiang Tung, Yishui Zhou, Iurii Kibalin, Bachir Ouladdiaf, Chao-Hung Du, Yixi Su

**Affiliations:** a Jülich Centre for Neutron Science JCNS at Heinz Maier-Leibnitz Zentrum (MLZ) Forschungszentrum Julich Lichtenbergstraße 1 Garching D-85747 Germany; b Department of Physics, Tamkang University, New Taipei City, 251301, Taiwan; c European Spallation Source (ERIC) PO Box 176 Lund SE-22100 Sweden; d Institut Laue–Langevin 71 avenue des Martyrs Grenoble 38042 France; Technical University of Denmark, Denmark

**Keywords:** Fe_3_GaTe_2_, van der Waals ferromagnets, single-crystal neutron diffraction, magnetic structure

## Abstract

High-quality single crystals of the two-dimensional van der Waals ferromagnet Fe_3_GaTe_2_ (FGaT) were successfully grown using the chemical vapour transport method, which effectively reduced surface impurities compared with conventional self-flux growth. Structural and magnetic characterizations were per­formed using single-crystal X-ray and neutron diffraction. The results confirm that FGaT crystallizes in the hexagonal *P*6_3_/*mmc* structure, with Fe occupying two inequivalent sites (Fe^i^ and Fe^ii^), where the magnetic moment of Fe^i^ [1.9 (2) μ_B_] is larger than that of Fe^ii^ [1.4 (6) μ_B_]. The magnetic easy axis is oriented along the *c* axis and the Curie temperature (*T*_C_) is approximately 355–360 K. Compared with Fe_3_GeTe_2_ (FGT), FGaT exhibits a slightly expanded *a* axis and a contracted *c* axis, resulting in a reduction in the Fe^i^–Fe^i^ interatomic distance along the *c* axis. This pronounced contraction could strengthen the Fe–Fe exchange interaction, which is believed to be the key factor responsible for the significantly higher *T*_C_ in FGaT relative to FGT.

## Abstract 2

High-quality single crystals of Fe_3_GaTe_2_ were grown using the chemical vapour transport method, allowing for a precise determination of their crystal and magnetic structures. A shortening of the Fe^i^–Fe^i^ distance enhances the Fe–Fe exchange interaction, providing a microscopic origin for its higher Curie temperature compared with Fe_3_GeTe_2_.

## 1. Introduction

In the field of spintronics, magnetic two-dimensional van der Waals (2D-vdW) materials have garnered considerable attention due to their potential applications, particularly in magnetic tunnel junctions and spin current transport, owing to their unique structures and magnetic anisotropy. As a result, they have become a frontier in contemporary condensed matter physics and materials science research. Fe_3_GaTe_2_ (FGaT) is a new type of 2D-vdW ferromagnet (Zhang *et al.*, 2022 ▸) with an extremely high Curie temperature (*T*_C_) of approximately 350–380 K, which has attracted numerous research teams over the past two years. Recent studies have explored various approaches to utilizing FGaT in spintronic devices, including achieving magnetic switching through current-induced spin-orbit torque (Li *et al.*, 2023*a* ▸; Kajale *et al.*, 2024 ▸; Zhang *et al.*, 2025 ▸), regulating *T*_C_ by applying a perpendicular electric field (Cai *et al.*, 2025 ▸) and manipulating interfacial electronic properties by forming heterostructures with different two-dimensional materials (Zhang *et al.*, 2024*b* ▸). In addition, it was reported that FGaT forms iron-atom defects easily, which produce spatial inversion symmetry breaking, leading to Dzyaloshinskii–Moriya interactions and ultimately formation of topologically protected skyrmions under external magnetic field induction (Li *et al.*, 2024 ▸; Zhang *et al.*, 2024*a* ▸). This means that FGaT has great potential for information storage and processing applications. Furthermore, because FGaT has a *T*_C_ above room temperature, the development of room-temperature magnetic 2D-vdW materials with novel magnetism through Co or Ni doping has also been studied (Son *et al.*, 2024 ▸; Yu *et al.*, 2024 ▸). These diverse applications are based on the combination of perpendicular magnetic anisotropy and high *T*_C_ in FGaT.

The atomic structure and magnetic properties of FGaT exhibit similarities to those of another related, but better-studied, 2D-vdW ferromagnet, Fe_3_GeTe_2_ (FGT). However, the *T*_C_ values of these two materials are significantly different, with the *T*_C_ of FGT being only 170–220 K, depending on the material defects (May *et al.*, 2016 ▸). Some studies try to explain the origin of the high *T*_C_ of FGT through first-principles calculations, focusing on the density of states near the Fermi level (Ruiz *et al.*, 2024 ▸; Wu *et al.*, 2024*b* ▸). Others have shown that changes in the lattice constant can cause internal stress or atomic displacement, thereby altering the exchange interaction and contributing to the enhancement of *T*_C_ (Lee *et al.*, 2023 ▸). All of these factors could lead to a higher *T*_C_ for FGaT than for FGT. However, some reports suggest that *T*_C_ might actually decrease when the FGT crystal structure is compressed under high pressure (Ding *et al.*, 2021 ▸; Dang *et al.*, 2023 ▸). In addition, some studies have shown that the intercalated Fe between van der Waals layers leads to enhanced magnetic interlayer coupling, which may be one of the reasons for the increase in *T*_C_ (Saha *et al.*, 2024 ▸; Lopez *et al.*, 2025 ▸). However, this phenomenon may also be caused by high-temperature pre-annealing (Zhou *et al.*, 2025 ▸) and therefore may not be present in all experiments.

The above studies all highlight the high *T*_C_ of FGaT, the cause of which remains a topic worthy of in-depth exploration. In this study, we grew single crystals of FGaT using chemical vapour transport (CVT) to avoid impurities in the sample. Single-crystal X-ray diffraction (SC-XRD) and single-crystal neutron diffraction were employed to investigate the differences in the atomic and magnetic structures of FGaT compared with FGT, with the aim of identifying the fundamental cause of the difference in *T*_C_ and contributing to the study of new room-temperature ferromagnetic 2D-vdW materials.

## 2. Experimental

High-quality single-crystal samples were produced using the CVT method. Lump Fe (99.99%), lump Ga (99.99%) and columnar Te (99.99%) were placed in a 14 cm-long quartz tube in an Ar-filled glove box in an atomic molar ratio of 3:1:2. A total of 5 g of raw material was added to the quartz tube, with iodine as a transport agent at approximately 2 mg cm^−3^ (Chen *et al.*, 2013 ▸; Tian *et al.*, 2019 ▸). The tube was sealed at an internal pressure of 1 × 10^−3^ mbar. To prevent iodine vaporization during the sealing process, the end of the tube was immersed in water for cooling. The furnace, with a hot zone of 1033 K and a cold zone of 983 K, was set for 168 h. FGaT single crystals were obtained after the reaction mixture was cooled naturally to room temperature. The sample quality was analysed using X-ray diffraction (XRD) data collected on a Bruker D2 PHASER diffractometer, which utilized Cu *K*α radiation (λ = 1.5406 Å) to examine the out-of-plane structural signals of the two-dimensional material. The magnetic susceptibility measurements were performed using a Quantum Design PPMS DynaCool system. For the χ–*T* measurements, zero-field cooling (ZFC) data were collected upon warming, while field cooling (FC) data were collected upon cooling. A DC magnetic field was applied at 2 K to measure the magnetization, which was then used to estimate the average magnetic moment per unit cell. SC-XRD data were collected using a Rigaku XtaLAB Synergy-S diffractometer (λ = 0.71073 Å), analysed with *CrysAlisPro* (Rigaku), and refined with *JANA2006* (Petříček *et al.*, 2014 ▸) and *FullProf* (Rodríguez-Carvajal, 1993 ▸) for atomic positions and occupancies. Neutron diffraction data were collected with a wavelength of 1.26 Å on the D10+ instrument at Institut Laue–Langevin (ILL), and the magnetic structure was refined using *FullProf*.

## 3. Results and discussion

**Figure 1:**
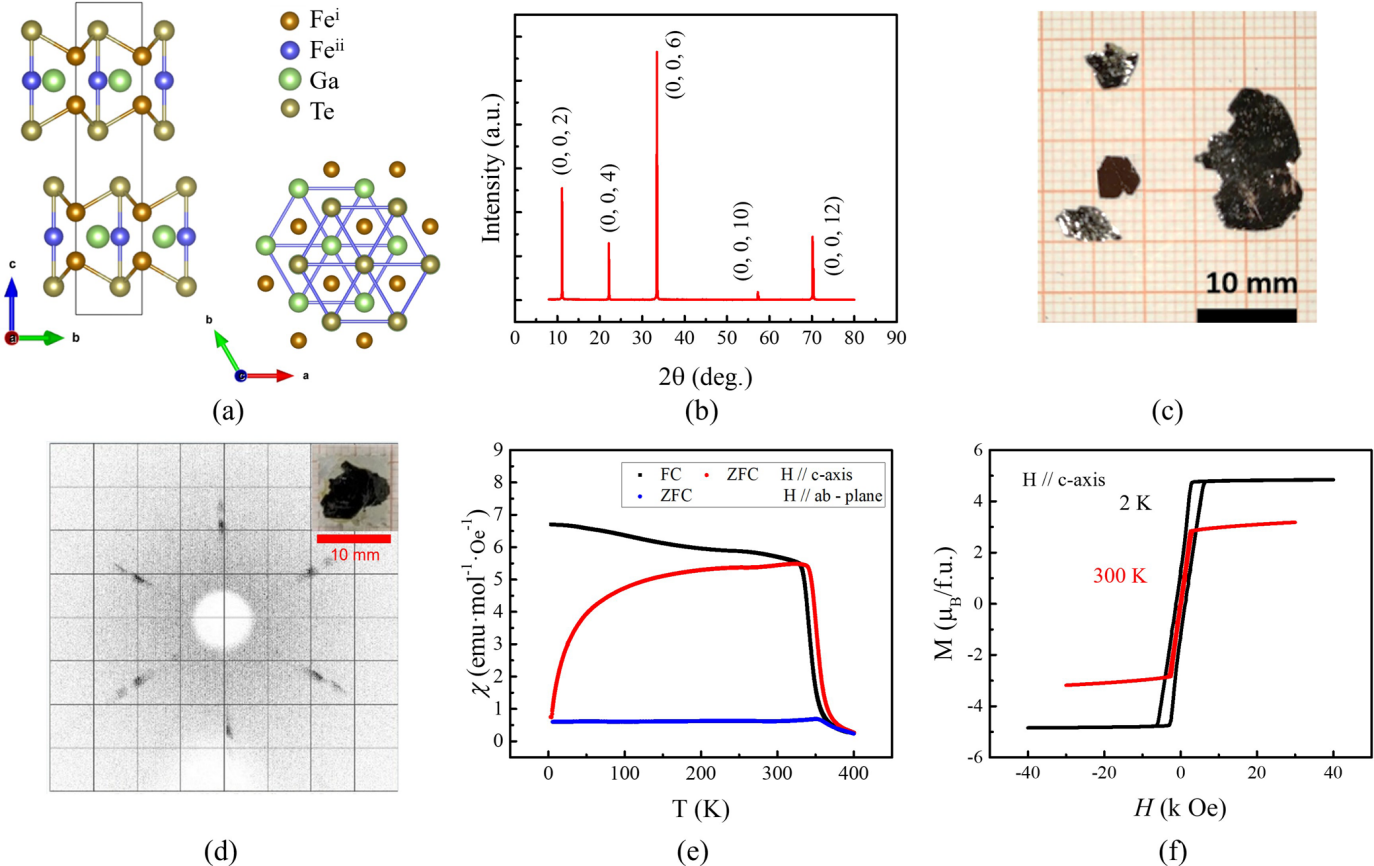
(*a*) Schematic diagrams of the side and top views of a van der Waals FGaT crystal. (*b*) XRD pattern along the normal face. (*c*) A photograph of the crystals as grown. (*d*) X-ray Laue image and (inset) photograph of the crystal used in neutron diffraction. (*e*) Temperature-dependent out-of-plane and in-plane magnetic susceptibility measurements under FC and ZFC conditions at 1000 Oe for bulk FGaT. (*f*) Out-of-plane magnetic hysteresis curves measured by vibrating sample magnetometry at 2 and 300 K.

The single crystals of FGaT grown using CVT exhibit a hexagonal layered crystal structure of space group *P*6_3_/*mmc*. Each van der Waals layer was formed by Te on the outside, with Fe_3_Ga layers sandwiched in between. Fe exists in two positions within the unit cell, denoted Fe^i^ and Fe^ii^. The van der Waals layers were stacked with their normal parallel to the *c* axis, similar to FGT [Fig. 1 ▸(*a*)]. Fig. 1 ▸(*b*) shows the XRD diffraction pattern of the freshly grown sample in the out-of-plane direction. All diffraction signals are from the (00*l*) planes of the two-dimensional material, with no extra diffraction, indicating a lack of surface impurities. On the other hand, if the self-flux method, as used in most reports, is employed to grow crystals, it is impossible to obtain crystals large enough and with low enough impurity content for a single-crystal neutron diffraction experiment. We initially attempted to grow crystals using the self-flux method in the molar ratio of Fe:Ga:Te = 1:1:2. However, the XRD diffraction pattern along the *c* axis revealed an additional diffraction peak at 26.2° (2θ) besides the reflections from the (00*l*) planes. This was probably from a Ga_2_Te_3_ alloy that had grown on the surface of the FGaT crystal, confirmed by energy-dispersive spectrometry (EDS). In contrast, EDS analysis of FGaT crystals grown via CVT showed a uniform elemental ratio of Fe:Ga:Te = 3:1:2 throughout the crystal. Therefore, both XRD along the *c* axis and EDS analysis confirmed that FGaT crystals grown using the CVT method were free of surface impurities.

Fig. 1 ▸(*c*) shows a photograph of the crystals as grown, while Fig. 1 ▸(*d*) shows a photograph of the sample used in this neutron single-crystal diffraction experiment and the corresponding X-ray Laue image. The temperature-dependent magnetic susceptibility in different directions, and the hysteresis loops measured at 2 and 300 K parallel to the *c* axis, are shown in Figs. 1 ▸(*e*) and 1 ▸(*f*), respectively. The small offset between the ZFC and FC curves is attributed to a slight difference in reaching thermal equilibrium of the sample between these two measurement protocols. The magnetic susceptibility measurements in different directions confirm that the magnetic easy axis was along the *c* axis, and *T*_C_ was estimated to be approximately 355–360 K using the Curie–Weiss law. The remanence of the hysteresis loop at 300 K was almost zero because the striped magnetic domains formed without the application of a magnetic field (Li *et al.*, 2024 ▸).

Due to the high sample purity, it was possible to calculate the average magnetic moment per Fe atom within the unit cell with the molar mass. From the magnetic moment values obtained in the saturation magnetic field region at 2 K, the average magnetic moment per Fe atom at 2 K was estimated to be approximately 1.76 μ_B_.

**Figure 2:**
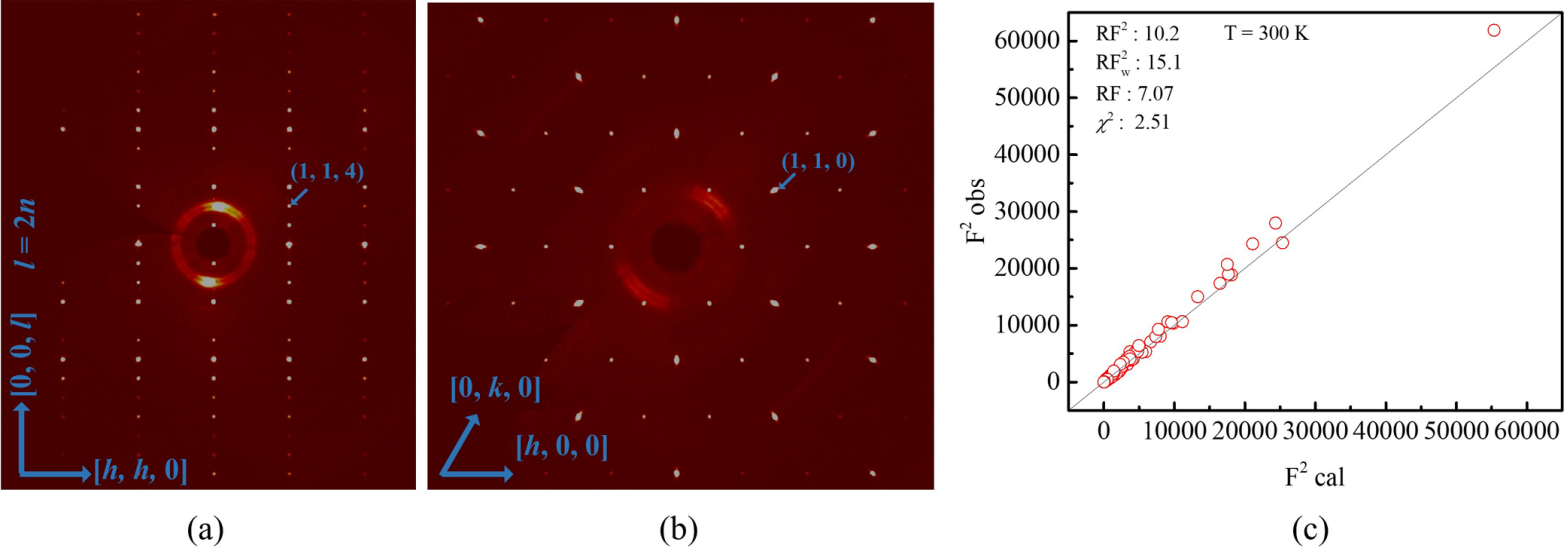
(*a*) SC-XRD along the [*hh*0] and [00*l*] directions. (*b*) SC-XRD along the [*h*00] and [0*k*0] directions confirms the crystal has hexagonal symmetry. (*c*) The refined nuclear structure result gives space group *P*6_3_/*mmc*.

SC-XRD measurements were used to analyse the structural details of FGaT. The unit-cell parameters of FGaT determined from the SC-XRD experiment are *a* = 4.0794 Å and *c* = 16.09040 (10) Å. A total of 411 reflections were used in the refinement. Previous studies have suggested that Fe atoms adjacent to Ga may undergo slight displacements along the *c* axis due to structural vacancies, leading to additional diffraction signals at *hhl* (where *l* = 2*n* + 1). These reflections are forbidden in the centrosymmetric space group *P*6_3_/*mmc* and therefore require the non-centrosymmetric space group *P*3*m*1 for structural description (Li *et al.*, 2024 ▸; Zhang *et al.*, 2024*b* ▸). However, in our single-crystal diffraction measurements [Fig. 2 ▸(*a*)], no *hhl* reflections with *l* = 2*n* + 1 were detected, indicating the absence of *P*3*m*1 symmetry. Thus, the crystal structure of our FGaT sample is best described by the *P*6_3_/*mmc* space group. The measured unit-cell parameters reveal that the *a* axis of FGaT is slightly longer than that of FGT, whereas the *c* axis is slightly shorter (Verchenko *et al.*, 2015 ▸).

The XRD refinement results of the nuclear structure are shown in Fig. 2 ▸(*c*). On the basis of the refinement results, we further analysed the occupancy of Fe atoms at different positions. Since it is generally believed in the literature that Ga atoms have no obvious defects, this study only focuses on Fe defects (Zhang *et al.*, 2022 ▸). Table 1 ▸ summarizes the refinement results of atomic positions and occupancy. Approximately 5% vacancy at the Fe^i^ site and 16% vacancy at the Fe^ii^ site were confirmed.

To analyse the magnetic structure of Fe_3_GaTe_2_, neutron single-crystal diffraction experiments were conducted on beamline D10+ at the ILL. To collect sufficient diffraction signals, diffraction peaks were collected from a 10 mg single crystal using a four-circle configuration of D10+ at 2 K and at room temperature. However, using the four-circle configuration comes at the cost of not being able to heat the sample above 360 K. Therefore, the subsequent refinement of the atomic structure and related information is based on data obtained from the XRD refinement.

**Figure 3:**
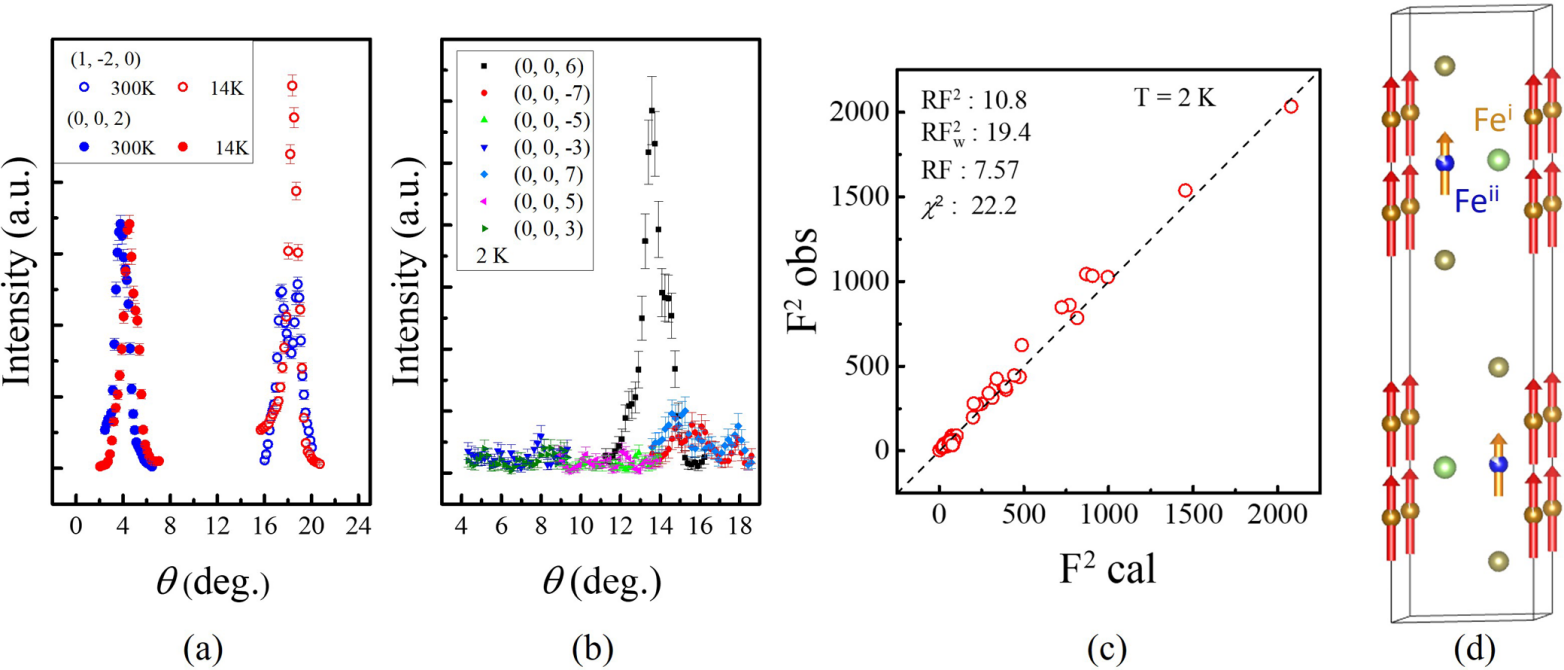
(*a*) The temperature dependence of the 002 and 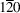 reflections in single-crystal neutron diffraction. (*b*) The diffraction signals of 00*l* (*l* = 2*n* + 1) and 006 at 2 K. (*c*) The refinement results of the magnetic structure with the atomic structure at 2 K. (*d*) The magnetic structure refinement result with the space group *P*6_3_/*mm*′*c*′. The magnetic moment of Fe^i^ is larger than that of Fe^ii^.

The crystal quality was initially assessed by recording Laue diffraction patterns [Fig. 1 ▸(*d*)], which confirmed that the sample is a single domain and possesses hexagonal symmetry. The diffraction intensities of 002 and 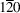 at 300 and 2 K are compared in Fig. 3 ▸(*a*). The intensity of 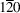 increases as the temperature is decreased, but the intensity of 002 does not, indicating that the components of the magnetic moment are indeed aligned along the *c* axis and have no components in the *ab* plane.

The diffraction signals of 00*l* at 2 K were used to confirm the symmetry of the structure. Fig. 2 ▸(*b*) shows the diffraction signals of 00*l* (*l* = 2*n* + 1) and 006 at 2 K for comparison. Although 006 is a very weak structural diffraction peak under neutron diffraction, no diffraction peaks of the odd-numbered 00*l* can be observed, confirming that no Fe^ii^ shift is observed under neutron diffraction.

Figs. 3 ▸(*c*) and 3 ▸(*d*) show the refinement results of the atomic and magnetic structures at 2 K. The previously published X-ray magnetic circular dichroism studies by Zha *et al.* (2023 ▸) identified Fe as the dominant source of magnetism in FGaT, while the contributions from Ga and Te are negligible. In addition, the magnetic susceptibility measurements indicate a ferromagnetic alignment of the magnetic moments along the *c* axis. On this basis, magnetic models with moments oriented along the *a* or *b* directions, as well as antiferromagnetic arrangements along the *c* axis, can be excluded.

To build the magnetic structure model, the *MAXMAGN* tool from the Bilbao Crystallographic Server was used (Aroyo *et al.*, 2006 ▸). After excluding those models that do not match experimental observations or which fail to converge after refinement, the magnetic structure was refined with the magnetic space group *P*6_3_/*mm*′*c*′ (No. 194.270), in which the magnetic moments of Fe^i^ and Fe^ii^ are parallel to each other and aligned along the *c* axis. In total, 57 reflections were used in the refinement. Because the structural parameters were fixed to the XRD results, only the scale factors and the magnetic moments on the Fe sites could be refined in the single-crystal neutron diffraction analysis. The average magnetic moment of 1.76 μ_B_ per Fe atom, based on our magnetic susceptibility measurement at 2 K, was considered in the magnetic structure refinement. In the final refinement, the scale factor was fixed so that the average magnetic moment of the two Fe sites, taking site occupancies into account, was consistent with the value obtained from the magnetic susceptibility measurements. Under this constraint, the refined magnetic moments are Fe^i^ = 1.9 (2) μ_B_ and Fe^ii^ = 1.4 (6) μ_B_. Therefore, the magnetic moments on the two Fe sites were not refined fully independently in the final model, and this constraint was applied to ensure consistency with the bulk magnetic measurements.

The magnetic moment of FGaT revealed by single-crystal neutron diffraction is similar to that of FGT, which was determined from a neutron powder diffraction analysis (Verchenko *et al.*, 2015 ▸). The two results are compared in Table 2 ▸. Many first-principles theoretical calculations have also obtained values close to these results (Yu *et al.*, 2024 ▸; Ruiz *et al.*, 2024 ▸; Zha *et al.*, 2023 ▸), indicating that the two magnetic structures are generally similar. However, the *T*_C_ of FGaT is much higher than that of FGT, indicating that the key factor affecting *T*_C_ may not come from the magnetic structure but rather be related to the electronic structure of each atom in the material, the strength of the exchange interaction and the difference in orbital overlap caused by changes in the lattice constant.

Other possible explanations for the high *T*_C_ of FGaT have also been studied. For example, each Fe site in Fe_5_GeTe_2_ has a different occupancy due to the thermal annealing processes used (Wu *et al.*, 2024*a* ▸), and a similar situation occurs in FGaT. Previous studies have shown that incorporating a small number of Fe atoms into the van der Waals gaps, along with antiferromagnetic coupling, yields simulated results that more closely match the neutron powder diffraction data (Lopez *et al.*, 2025 ▸). These interlayer Fe atoms were thus suggested as a possible origin of the enhanced *T*_C_ (Lopez *et al.*, 2025 ▸). However, it was subsequently reported that the formation of such intercalated Fe can be induced by annealing. Even in the absence of intercalated Fe atoms, the *T*_C_ of FGaT is still much higher than room temperature (Zhou *et al.*, 2025 ▸). Therefore, intercalated Fe should not be the main reason for the high *T*_C_ phenomenon. In addition, since our sample was not repeatedly annealed after growth, the case of Fe intercalation was not a concern.

Density functional theory (DFT) has been used to confirm clearly that the magnetism of the FGT system comes from local magnetic moments and itinerant electrons (Bao *et al.*, 2022 ▸; Xu *et al.*, 2024 ▸), so it can be considered a typical Hund metal. Although FGaT also exhibits the coexistence of localization and itinerant character, its overall magnetism is more clearly dominated by local magnetic moments contributed by Fe^i^ (Wu *et al.*, 2024*b* ▸; Xu *et al.*, 2025 ▸). Lee *et al.* (2023 ▸) further demonstrated that the reduction in the Fe–Fe interatomic distance enhances the overlap between Fe 3*d* orbitals, leading to a substantial increase in the nearest-neighbour Heisenberg exchange constant *J*_1_. This strengthened exchange interaction is considered to be a key factor in the higher *T*_C_ observed in FGaT relative to FGT.

Based on the results of the SC-XRD refinement, a comparative analysis was conducted between the crystal structures of FGT and FGaT. The refined interatomic distances for FGaT are summarized in Table 3 ▸, while the corresponding parameters for FGT can be found in previous reports on its atomic and magnetic structure (Verchenko *et al.*, 2015 ▸; included in Table 3 ▸ for comparison). The refinement results show that FGaT expands slightly along the *a* axis and contracts slightly along the *c* axis, resulting in slight changes in the interatomic spacings compared with FGT. Notably, the Fe^i^–Fe^i^ distance along the *c* axis in FGaT exhibits the largest variation compared with that in FGT. Such a pronounced contraction is expected to strengthen the Fe^i^–Fe^i^ exchange interaction and enhance the nearest-neighbour Heisenberg exchange-coupling interaction, thereby increasing the thermal energy required to destabilize the ordered magnetic state.

To place this structural difference in a clear physical context, a comparison between FGT and FGaT was performed using reported experimental and theoretical results. In the work of Ghosh *et al.* (2023 ▸) on FGT, first-principles DFT calculations via GGA+DMFT showed that, for an Fe^i^–Fe^i^ distance of 2.47 Å along the *c* axis, the nearest-neighbour exchange interaction parameter *J*_1_ is approximately 37 meV. In contrast, Lee *et al.* (2023 ▸) reported that for FGaT a shorter Fe^i^–Fe^i^ distance of 2.409 Å along the *c* axis leads to an increased exchange parameter *J*_1_ = 74.83 meV. Experimentally, Verchenko *et al.* (2015 ▸) reported an Fe^i^–Fe^i^ distance of 2.602 Å for FGT, whereas in our present work, the Fe^i^–Fe^i^ distance along the *c* axis is refined to be 2.479 Å for FGaT. Ghosh *et al.* (2023 ▸) further showed that when only GGA is used (without DMFT) the exchange parameter *J*_1_ in FGT increases to 65.32 meV, yielding an estimated *T*_C_ of approximately 470 K. These results indicate that a reduction in the Fe^i^–Fe^i^ distance can indeed lead to an increase in the exchange parameters and consequently to an enhancement of *T*_C_. However, the quantitative relationship between the distance contraction and the exact change in the exchange parameter remains highly model dependent. For example, Li and co-workers reported an Fe^i^–Fe^i^ distance of 2.37 Å with an exchange parameter *J*_1_ = 57.18 meV, illustrating that substantial variations may exist among different theoretical studies. On the basis of these comparisons, we conclude that the Fe^i^–Fe^i^ distance in FGaT is shorter than that in FGT by approximately 0.12 Å (Verchenko *et al.*, 2015 ▸), and that this reduction corresponds, according to various DFT calculations, to an increase in the exchange parameter *J*_1_ of the order of roughly 20–40 meV (Lee *et al.*, 2023 ▸; Li *et al.*, 2023*b* ▸). Such a substantial increase is sufficient to account for an enhancement of *T*_C_ from approximately 220–230 K to 350–380 K, consistent with the experimental observations.

Regarding the relative roles of the Ga site and the Fe^ii^ site in influencing the interlayer Fe^i^–Fe^i^ distance, this issue can be addressed by comparing the structural differences between FGaT and FGT, as well as the effect of Fe^ii^ vacancies in FGT. For FGT, the lattice parameters are *a* = 4.00848 (2) Å and *c* = 16.3307 (1) Å (Verchenko *et al.*, 2015 ▸). In contrast, our SC-XRD measurements on FGaT yield *a* = 4.0794 Å and *c* = 16.09040 (10) Å, in excellent agreement with previously reported values [*a* = 4.09 (2) Å and *c* = 16.07 (2) Å; Lee *et al.*, 2023 ▸]. This comparison suggests that substitution of Ge by Ga leads primarily to a shortening of the *c* axis and a slight expansion of the *a* axis, resulting in a reduced Fe^i^–Fe^i^ distance. In contrast, May *et al.* (2016 ▸) showed that in Fe_3−*x*_GeTe_2_ increasing the Fe^ii^ vacancy concentration led to a decrease in the lattice parameter *a* and an increase in *c*, accompanied by a reduction in *T*_C_. Similarly, Bao *et al.* (2022 ▸) reported that defective FGT exhibits a reduced *T*_C_ down to approximately 160 K. These observations indicate that Fe^ii^ vacancies do not promote a contraction of the Fe^i^–Fe^i^ interlayer distance. Instead, they tend to have the opposite effect. Therefore, the Fe^i^–Fe^i^ distance reduction observed in FGaT is most reasonably attributed to Ga/Ge substitution rather than to Fe^ii^ vacancies. Furthermore, the contraction of the *c* axis would normally be expected to shorten the Te–Fe^i^ distances. However, the excessive reduction in the Fe^i^–Fe^i^ spacing instead results in a slight elongation of the Te–Fe^i^ bond. The influence of Te on the Fe electronic states remains largely unexplored and requires further investigation.

## 4. Conclusions

In this study, we have demonstrated that the CVT method enables the growth of higher-quality FGaT single crystals compared with using the self-flux method, thereby benefitting single-crystal diffraction experiments. SC-XRD and neutron diffraction measurements have been employed to investigate the atomic and magnetic structures of FGaT, confirming that the magnetic moment of Fe^i^ is larger than that of Fe^ii^.

After replacing Ge with Ga, the van der Waals layered structure becomes slightly thinner and wider, and there is a significant shortening of the Fe^i^–Fe^i^ distance along the *c* axis. This structural modification provides important insight into the physical reasons for the significantly higher *T*_C_ observed in FGaT than in FGT.

## Supplementary Material

Crystal structure: contains datablock(s) global, I. DOI: 10.1107/S160057672600230X/chn5005sup1.cif

CCDC reference: 2534687

## Figures and Tables

**Table 1:** Atom parameters from SC-XRD of FGaT

Atom	Site	*x*	*y*	*z*	Occupancy	*B*_iso_ (Å^2^)
Fe^i^	4*e*	0	0	0.67296 (8)	0.95 (1)	0.839 (31)
Fe^ii^	2*d*	2/3	1/3	3/4	0.841 (16)	0.732 (46)
Ga	2*c*	1/3	2/3	3/4	1	1.665 (35)
Te	4*f*	2/3	1/3	0.59053 (4)	1	1.032 (16)

**Table 2:** Comparison of the magnetic moments of FGaT and FGT The results for FGT are based on neutron powder diffraction (Verchenko *et al.*, 2015 ▸).

Parameter	FGaT	FGT
*M* (Fe^i^) (μ_B_)	1.9 (2)	1.95 (5)
*M* (Fe^ii^) (μ_B_)	1.4 (6)	1.56 (4)

**Table 3:** Interatomic distances of FGaT from SC-XRD, compared with reported values for FGT (Verchenko *et al.*, 2015 ▸)

Bond		Distance in FGaT (Å)	Distance in FGT (Å)
Ga/Ge	Fe^i^	2.6615 (7)	2.655
	Fe^ii^	2.35524 (0)	2.314
Fe^i^	Fe^i^	2.479 (3)	2.602
	Fe^ii^	2.6615 (7)	2.655
	Ga	2.6615 (7)	2.655
	Te	2.7030 (8)	2.611
Fe^ii^	Ga	2.35524 (0)	2.314
	Te	2.5659 (7)	2.613
	Fe^i^	2.6615 (7)	2.655

## Data Availability

Neutron diffraction data from D10+ (ILL, France) are available at https://doi.ill.fr/10.5291/ILL-DATA.5-41-1243.
